# Construction and validation of a prognostic risk model for breast cancer based on protein expression

**DOI:** 10.1186/s12920-022-01299-5

**Published:** 2022-07-04

**Authors:** Bo Huang, Xujun Zhang, Qingyi Cao, Jianing Chen, Chenhong Lin, Tianxin Xiang, Ping Zeng

**Affiliations:** 1 Department of Gynecology and Obstetrics, The First Affiliated Hospital, Zhejiang University School of Medicine, Hangzhou, China; 2 State Key Laboratory for Diagnosis and Treatment of Infectious Diseases National Clinical Research Center for Infectious Diseases, National Medical Center for Infectious Diseases, Collaborative Innovation Center for Diagnosis and Treatment of Infectious Diseases, The First Affiliated Hospital, Zhejiang University School of Medicine, Hangzhou, China; 3 Department of Gastroenterology, Sir Run Run Shaw Hospital, Zhejiang University School of Medicine, Hangzhou, China; 4 Department of Hospital Infection Control, The First Affiliated Hospital of Nanchang University, 17 Yongwai Road, Donghu District, Nanchang, China

**Keywords:** Breast cancer, Proteomics, TCPA, TCGA, Prognostic risk model

## Abstract

Breast cancer (BRCA) is the primary cause of mortality among females globally. The combination of advanced genomic analysis with proteomics characterization to construct a protein prognostic model will help to screen effective biomarkers and find new therapeutic directions. This study obtained proteomics data from The Cancer Proteome Atlas (TCPA) dataset and clinical data from The Cancer Genome Atlas (TCGA) dataset. Kaplan–Meier and Cox regression analyses were used to construct a prognostic risk model, which was consisted of 6 proteins (CASPASE7CLEAVEDD198, NFKBP65-pS536, PCADHERIN, P27, X4EBP1-pT70, and EIF4G). Based on risk curves, survival curves, receiver operating characteristic curves, and independent prognostic analysis, the protein prognostic model could be viewed as an independent factor to accurately predict the survival time of BRCA patients. We further validated that this prognostic model had good predictive performance in the GSE88770 dataset. The expression of 6 proteins was significantly associated with the overall survival of BRCA patients. The 6 proteins and encoding genes were differentially expressed in normal and primary tumor tissues and in different BRCA stages. In addition, we verified the expression of 3 differential proteins by immunohistochemistry and found that CDH3 and EIF4G1 were significantly higher in breast cancer tissues. Functional enrichment analysis indicated that the 6 genes were mainly related to the HIF-1 signaling pathway and the PI3K-AKT signaling pathway. This study suggested that the prognosis-related proteins might serve as new biomarkers for BRCA diagnosis, and that the risk model could be used to predict the prognosis of BRCA patients.

**Supplementary Information:**

The online version contains supplementary material available at 10.1186/s12920-022-01299-5.

## Introduction

Breast cancer is the most common malignant tumor in women and ranks as the leading cause of cancer-related death in women, accounting for 15.3% of all cancer deaths among females. In 2020, according to the latest data released by the International Agency for Research on Cancer (IARC) of the World Health Organization (WHO) (http://www.irac.fr), there were 19.29 million new cancer patients, of which 2.26 million were breast cancer, replacing lung cancer as the leading cause of cancer [1]. One of the underlying causes of the increased incidence of breast cancer is the changing risk factors, such as delayed and reduced childbearing, overweight and obesity, lifestyle, and heredity. The prognosis of patients with breast cancer depends on the tumor stage. The five-year survival rate for early breast cancer is close to 100%, while the survival rate for advanced-stage patients remains poor [2]. Therefore, the early screening and diagnosis of breast cancer are critical. As the gold standard for the early detection of breast cancer, the screening modality of mammography is sensitively for fatty breast tissue and contributes to significantly reduced mortality [3]. However, mammography is not particularly useful for dense breast tissue. Studies indicate that women with dense tissue have a 4- to 6-fold greater risk of developing breast cancer than those with no dense tissue in the breast [4, 5]. The automated breast ultrasound (ABUS) screening system designed for dense breast tissue perfectly addressed the limitations of mammography [6]. Although breast cancer screening can assist diagnosis and treatment, yet it has several limitations. A frequent limitation is a false-positive screening outcome, leading to overdiagnosis, which may cause distress and anxiety in women [7]. In addition, screening cannot detect all breast cancers. Therefore, it is very important to search for more accurate and sensitive diagnostic and prognostic biomarkers.

To date, some proteins and RNAs have been proved to be prognostic factors for cancer. Common serum protein tumor markers, such as CEA, AFP, CA-125, and CA-199, have been widely used for the diagnosis and treatment of various cancer types. Carcinoembryonic antigen (CEA) originates from the cavitary organs and has relatively high sensitivity for adenocarcinomas [8]. CEA is the most extensively used tumor marker, including in lung adenocarcinoma, colorectal cancer, and gastric cancer [9–11]. Serum α-fetoprotein (AFP) is the most classic diagnostic marker in hepatocellular carcinoma (HCC) [12]. Cancer antigen 125 (CA125) is a traditional marker for ovarian cancer screening [13] and is insufficient to diagnose ovarian cancer due to a lack of specificity [14]. Carbohydrate antigen 199 (CA199) is the most sensitive tumor marker for pancreatic cancer [15]. Squamous cell carcinoma antigen (SCC-Ag) is the most common early tumor marker for cervical cancer [16]. In addition, a growing number of studies have reported the potential of different RNAs as diagnostic biomarkers in cancer. The long noncoding RNA CCEPR (cervical carcinoma expressed PCNA regulatory) is a potential prognostic biomarker and therapeutic target [17]. Plasma miR-21 and miR-222 were increased in patients with gastric cancer and could be potential diagnostic biomarkers of gastric cancer [18]. miR1246 is a biomarker in esophageal and gastric cancers [19].

In human breast cancer, cathepsin D (CTSD), IL4 receptor (IL4R), mucin-1 (MUC1, CD227), and serine protease 3 (PRSS3) may serve as valuable biomarkers for the diagnosis and treatment of breast cancer [20–23]. Breast cancer is divided into different types based on pathology features. A single protein or RNA is unlikely to fulfill the criteria for identifying all types of breast cancer, resulting in missed diagnosis and overdiagnosis. Therefore, prognostic models based on multiple protein biomarkers have great potential for the diagnosis and prognosis of patients with breast cancer.

In this study, we downloaded the protein expression profile of breast invasive carcinoma (BRCA) from the Cancer Proteome Atlas (TCPA) database, and based on the Kaplan–Meier method and Cox regression analysis, we constructed a prognostic risk model for BRCA.

## Materials and methods

### Protein data acquisition and processing

The Cancer Proteome Atlas (TCPA) database provides the protein expression profiles for a variety of human cancers by integrating reversed-phase protein array (RPPA) chip data from The Cancer Genome Atlas (TCGA) database and several independent tumor research projects [24]. The TCPA database contains two separate web applications, one of which is the RPPA data for the patients with tumors, containing approximately 8000 cancer samples from 32 cancer types in the TCGA database, and another approximately 500 samples from an independent patient cohort. We downloaded the level 4 dataset of the BRCA protein expression profile from the TCPA database (http://www.tcpaportal.org/tcpa/) [25]. Matching clinical information of BRCA patients was downloaded from the TCGA database (http://portal.gdc.cancer.gov/), which is the currently largest cancer genetic information database. The missing data of the protein expression profiles were filled in with the “impute” package in the R software. Survival information was extracted from the clinical data and merged with the protein expression profile using Perl software.

### Screening the prognosis-related proteins

Kaplan–Meier (KM) survival analysis and univariate Cox analysis were used to screen the prognosis-related protein by the “survival” package in R software [26]. The patients were divided into two groups according to the protein expression and the KM method was used to analyze the difference of the two groups (*p* value < 0.05). The COX method compared protein expression as a continuous variable with survival time to observe whether there was a correlation (*p* value < 0.05). R packages “ggplot2” and “ggrepel” were used to draw the volcano map of differently expressed proteins [27]. Proteins with Hazard ratio (HR) < 1were considered as low-risk proteins, while proteins with HR > 1 were considered high-risk proteins.

### Constructing the protein prognostic risk model

The proteins with statistical difference were analyzed by multivariate Cox analysis and to build a prognostic risk model with the “survival” package in R software [28]. The coefficients of each protein in the model and the risk values of all samples were obtained. The risk score formula was defined as follows: risk score = (Coefficient Protein1 × expression of Protein1) + (Coefficient Protein2 × expression of Protein2) + ⋯ + (Coefficient Protein*n* × expression of Protein*n*) [29]. Patients were divided into high risk group and low risk group according to the median of the risk value.

### Assessing the protein prognostic risk model

The survival analysis of protein expression and risk values was conducted by the “survminer” and “survival” packages in R software. Risk curves, including the risk score, survival time, and protein expression, were performed using the “pheatmap” package. An independent prognostic analysis was using Perl software and the “survival” package based on the risk score and clinical data of each sample. The age, pathology stage, tumor (T), metastasis (M), node (N) states, and riskScore of the sample were considered by univariate and multivariate Cox analyses. The receiver operating characteristic (ROC) curve was analyzed by the “survivalROC” package in R software.

### Validating the protein prognostic risk model

Microarray datasets, including gene expression profiles and corresponding clinical information data of GSE88770, were downloaded from the Gene Expression Omnibus database (GEO, https://www.ncbi.nlm.nih.gov/geo/). GSE88770 was conducted by GPL570 (Affymetrix Human Genome U133 Plus 2.0 Array), including 117 breast cancer samples that were enrolled in our testing dataset. The expression profiles of mRNAs from GEO are shown as raw data and each mRNA was normalized by log2 transformation for further analysis.

### Expression of prognosis-related proteins and their encoding genes

The UALCAN portal (http://ualcan.path.uab.edu/analysis-prot.html) analyzed the expression of prognosis-related proteins and their encoding genes using the Clinical Proteomic Tumor Analysis Consortium (CPTAC) dataset (http://proteomics.cancer.gov/programs/cptac) and TCGA database. The immunohistochemical images of these proteins were obtained from the Human Protein Atlas (https://www.proteinatlas.org/).

### Functional enrichment analysis

The functional enrichment analysis was constructed using the Database for Annotation, Visualization, and Integrated Discovery (DAVID) Bioinformatics Resources 6.8 (https://david.ncifcrf.gov/home.jsp) including Gene Ontology (GO) analysis and Kyoto Encyclopedia of Genes and Genomes (KEGG) pathway enrichment analysis [30–34]. We used the bioinformatics online tool (http://www.bioinformatics.com.cn) to display the result.

### Immunohistochemistry

Immunohistochemistry was performed as standard protocols. Tissue sections of tumor and para-tumor tissues underwent dewaxing, hydration, antigen repair, and blocking, the sections were incubated overnight at 4 °C in primary antibody (anti-CDH3, ab242060, Abcam; anti-CASP7, ab255818, Abcam; anti-EIF4G1, 15704-1-AP, Proteintech.). After rinsing in phosphate-buffered saline (PBS) three times, the slides were incubated with a secondary antibody and the streptavidin–horseradish peroxidase in turn. At last, the slides were stained with DAB solution. Images were obtained using a direct optical microscope.

### Statistical analysis

R software (version 3.5.1) or Perl software (Strawberry Perl 5.30.0.1 64-bit) was performed analyses and chart visualize in this study. Statistical analysis of GSE88770 dataset was performed by using GraphPad Prism version 8.0 or SPSS version 19.0 software package. A two-tailed *p* < 0.05 was considered statistically significant.

## Results

### Screening prognostic-related proteins and constructing a prognostic risk model

**Fig. 1:**
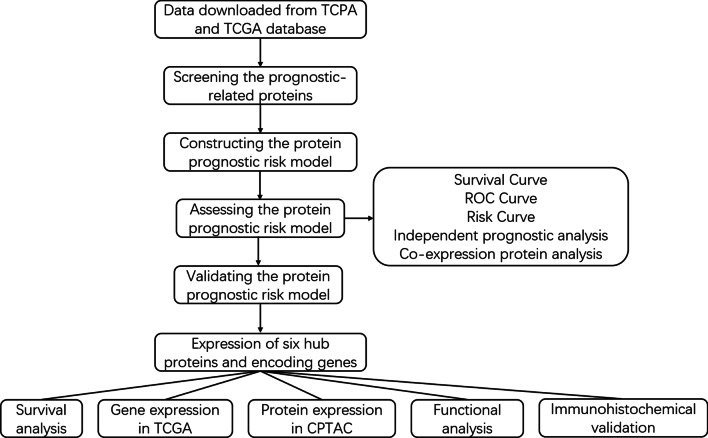
The flow diagram of this study

**Fig. 2:**
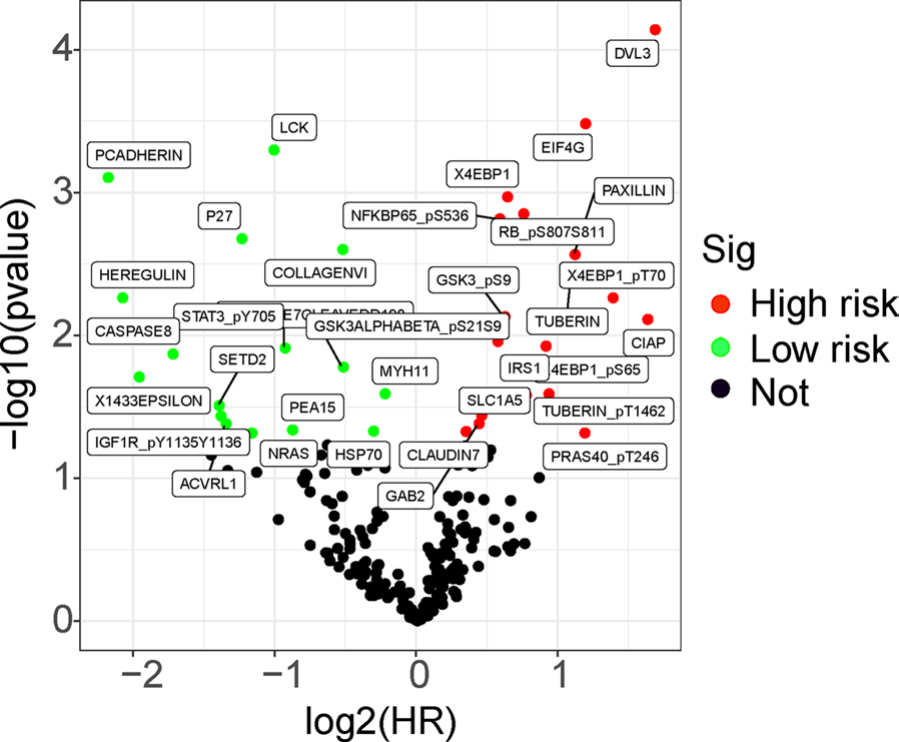
Volcano plot showing the differently expressed proteins in BRCA

The major work of this study is shown in Fig. 1. The protein expression profile of BRCA from the TCPA database and clinical data from the TCGA database were processed and analyzed. Univariate Cox regression analysis and the KM method identified 34 proteins that were significantly associated with the survival of BRCA patients, including 18 high risk proteins (hazard ratio > 1) and 16 low-risk proteins (hazard ratio < 1). All the significantly expressed proteins are displayed in a volcano plot (Fig. 2, Additional file 1: Table S1). Diahevelled-3 (DVL3, HR = 3.206, 95% CI = 1.803–5.702) was the highest-risk protein, while placental-cadherin (PCADHERIN, HR = 0.220, 95% CI = 0.091–0.533) was the lowest-risk protein. Multivariate Cox regression analysis demonstrated that six proteins were used to construct the prognostic model, including CASPASE7CLEAVEDD198, NFKBP65-pS536, PCADHERIN, P27, X4EBP1-pT70, and EIF4G. The formula for the risk model was as follows: Risk Score = CASPASE7CLEAVEDD198 * (− 0.406) + NFKBP65-pS536 * 0.242 + PCADHERIN * (− 1.157) + P27 * (− 0.640) + X4EBP1-pT70 * 0.710 + EIF4G * 0.534. These proteins were expressed in all samples and risk values for all patients were calculated based on this formula.

### Assessing of the protein prognostic risk model

**Fig. 3:**
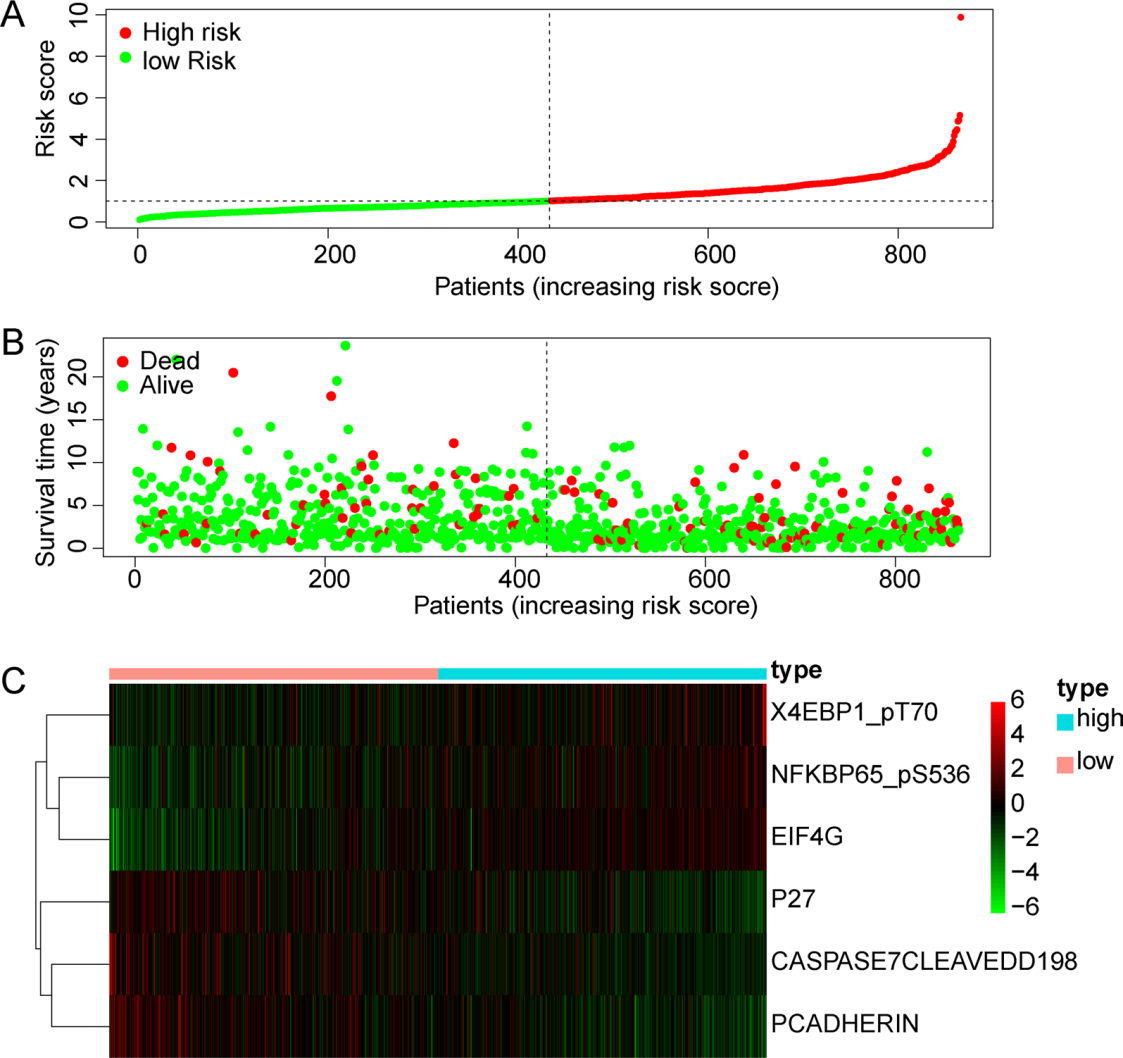
Construction of a protein prognostic risk model in BRCA. **A** The distribution of risk score of the BRCA patients. **B** The survival status of the patients based on the risk score. **C** The expression of the six proteins between the high-risk group and the low risk group

**Fig. 4:**
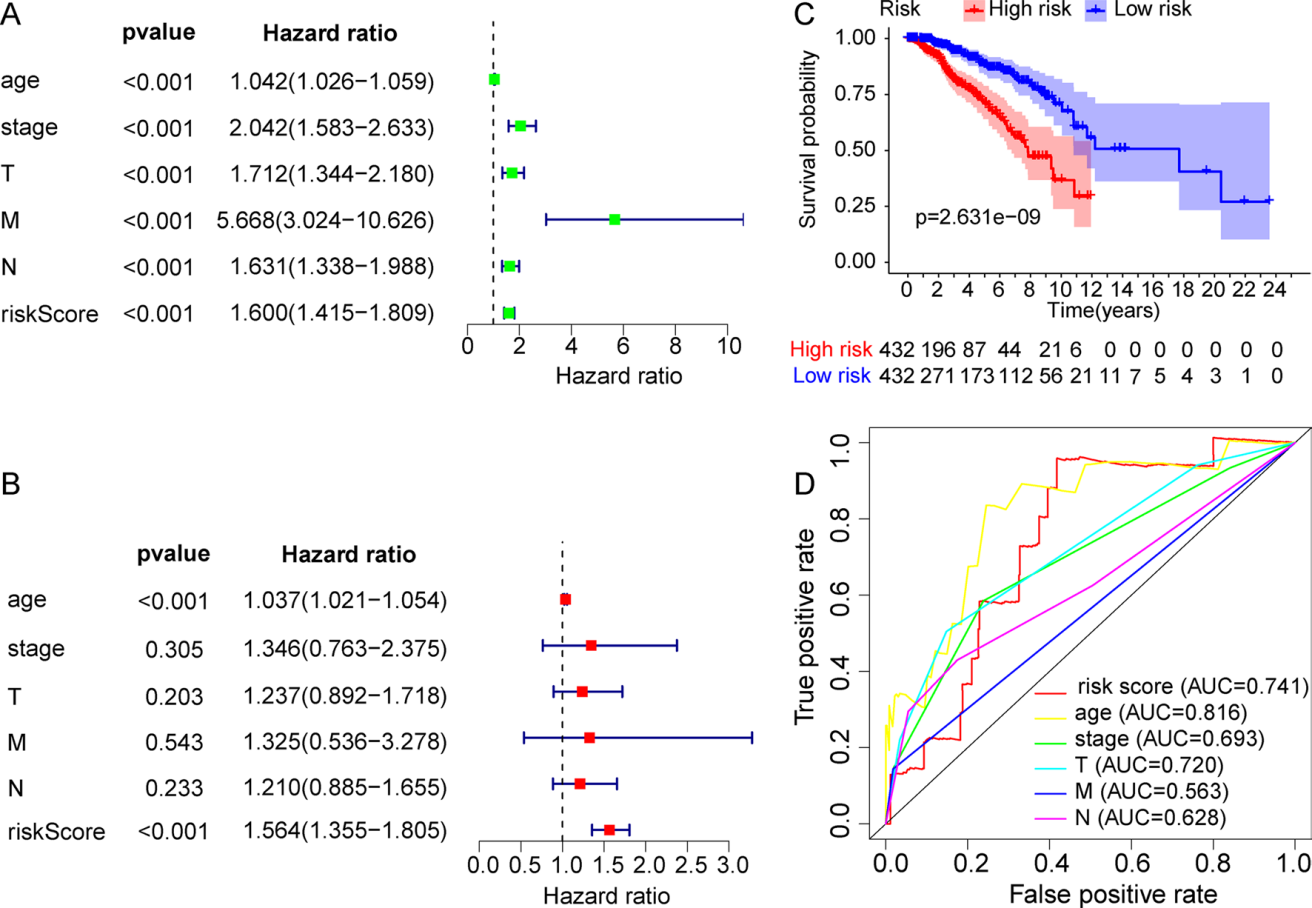
Assessment of the prognostic risk model in the BRCA patients. **A** Univariate Cox regression analysis was performed to assess the prognostic model. **B** Multivariate Cox regression analysis was performed to assess the prognostic model. **C** The overall survival analysis of the BRCA patients. **D** Receiver operating characteristic (ROC) curve revealed the performance of the prognostic risk model in BRCA

To further develop a comprehensive prognostic risk model, we built a risk curve, including risk score, survival time, and protein expression. The risk score distribution of the BRCA patients is shown in Fig. 3A. The higher the risk score, the worse the survival state (Fig. 3B). The heatmap displays the expression profiles of the 6 proteins in the high-risk and low-risk groups. EIF4G (EIF4G1), NFKBP65-pS536 (RELA), and X4EBP1-pT70 (EIF4EBP1) were highly expressed in the high-risk score group, while CASPASE7CLEAVEDD198 (CASP7), P27 (CDKN1B), and PCADHERIN (CDH3) were highly expressed in the low-risk score group (Fig. 3C). To evaluate whether the prognostic model was independent of other clinical factors, we performed univariate and multivariate Cox regression analyses. We found that clinical traits were associated with survival time and survival state, including age, pathology, T stage, M stage, N stage, and risk score, by univariate Cox regression analysis. The clinical characteristics of BRCA patients were shown in Additional file 1: Table S2. Univariate Cox analysis demonstrated that the risk score (HR 1.600 (1.415–1.809), *p* < 0.001), age (HR 1.042 (1.026–1.059), *p* < 0.001), stage (HR 2.042 (1.583–2.633), *p* < 0.001), T (HR 1.712 (1.344–2.180), *p* < 0.001), M (HR 5.668 (3.024–10.626), *p* < 0.001), N (HR 1.631(1.338–1.988), *p* < 0.001). Multivariate Cox regression analysis again suggested that risk score (HR 1.564 (1.355–1.805), *p* < 0.001) and age (HR 1.037 (1.021–1.054), *p* < 0.001) could be viewed as independent prognostic factors for BRCA patients (Fig. 4A, B). In addition, we divided all samples into high- and low-risk groups according to the median risk value of each sample. Overall survival analysis showed that survival probability and survival time were significantly decreased in the high-risk group compared with the low risk group (Fig. 4C). The ROC curve could evaluate the accuracy of the prognostic model in predicting the survival time of the BRCA patients. The area under the curve (AUC) of the risk score was 0.741, suggesting that the predictive effectiveness was sensitive and significant for the prognostic risk model (Fig. 4D). These results suggested that this prognostic model could be viewed as an independent factor to accurately predict the survival time of BRCA patients.

### Validation of the protein prognostic risk model

**Fig. 5:**
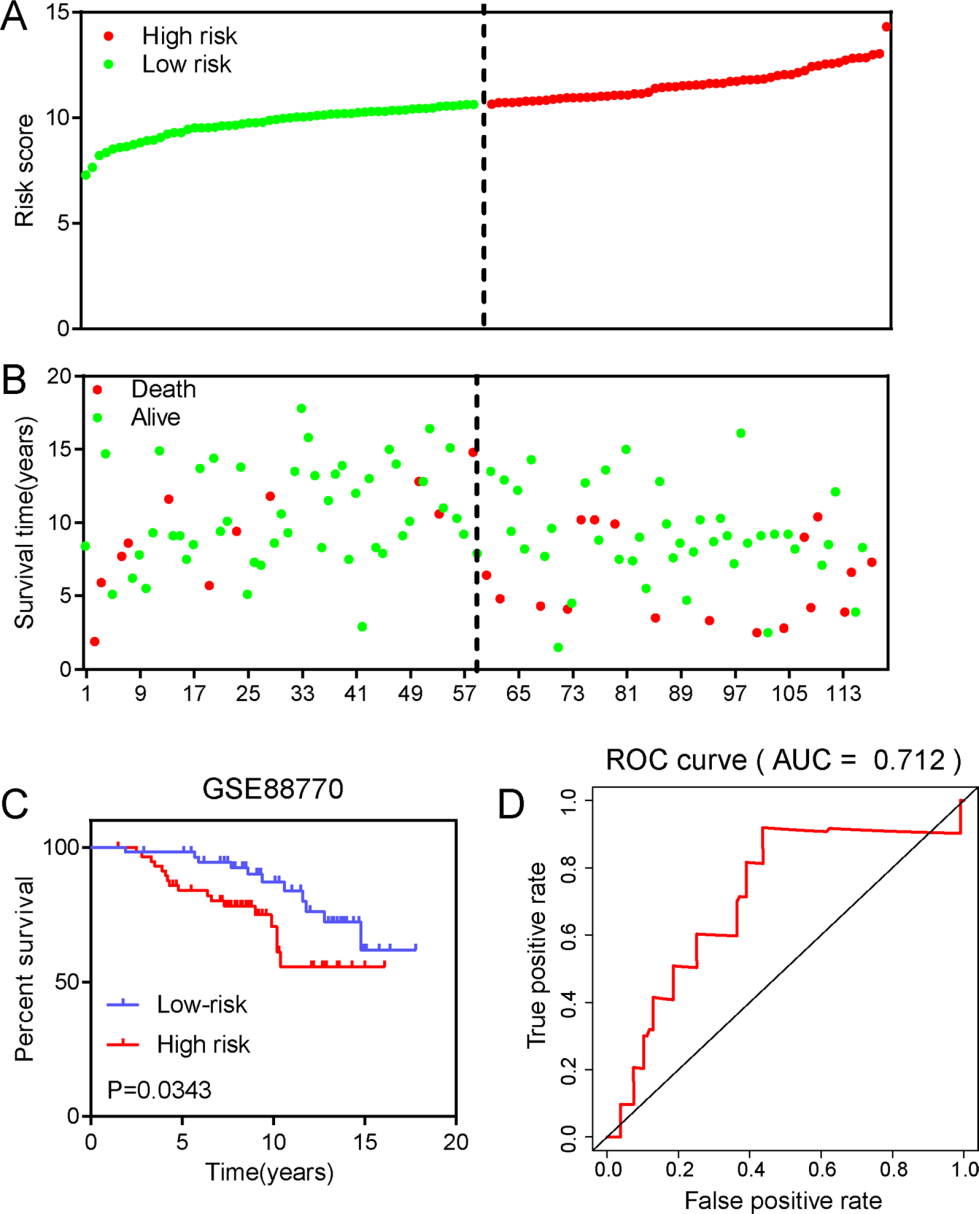
Validation of a protein prognostic risk model in BRCA. **A** The distribution of risk score. **B** The survival status of the patients based on the risk score. **C** The overall survival analysis of the BRCA patients. **D** The ROC curve of the prognostic risk model in GSE88770 dataset

To demonstrate the prognostic performance and a possible future application of this model, we downloaded the GSE88770 dataset and validated the prognostic model in the microarray datasets. The clinical characteristics of BRCA patients were shown in Additional file 1: Table S3. The results showed that higher risk scores indicated a shorter survival time (Fig. 5A, B). Similarly, we found significantly higher survival rates in the low-risk group than in the high-risk group by overall survival analysis (Fig. 5C). The AUC of the risk score in the GSE88770 dataset was 0.712 indicating good predictive ability of the prognostic risk model (Fig. 5D). The above results validated that this prognostic model had good predictive performance and could be used to predict the risk of BRCA patients.

### Survival analysis of the 6 proteins

**Fig. 6:**
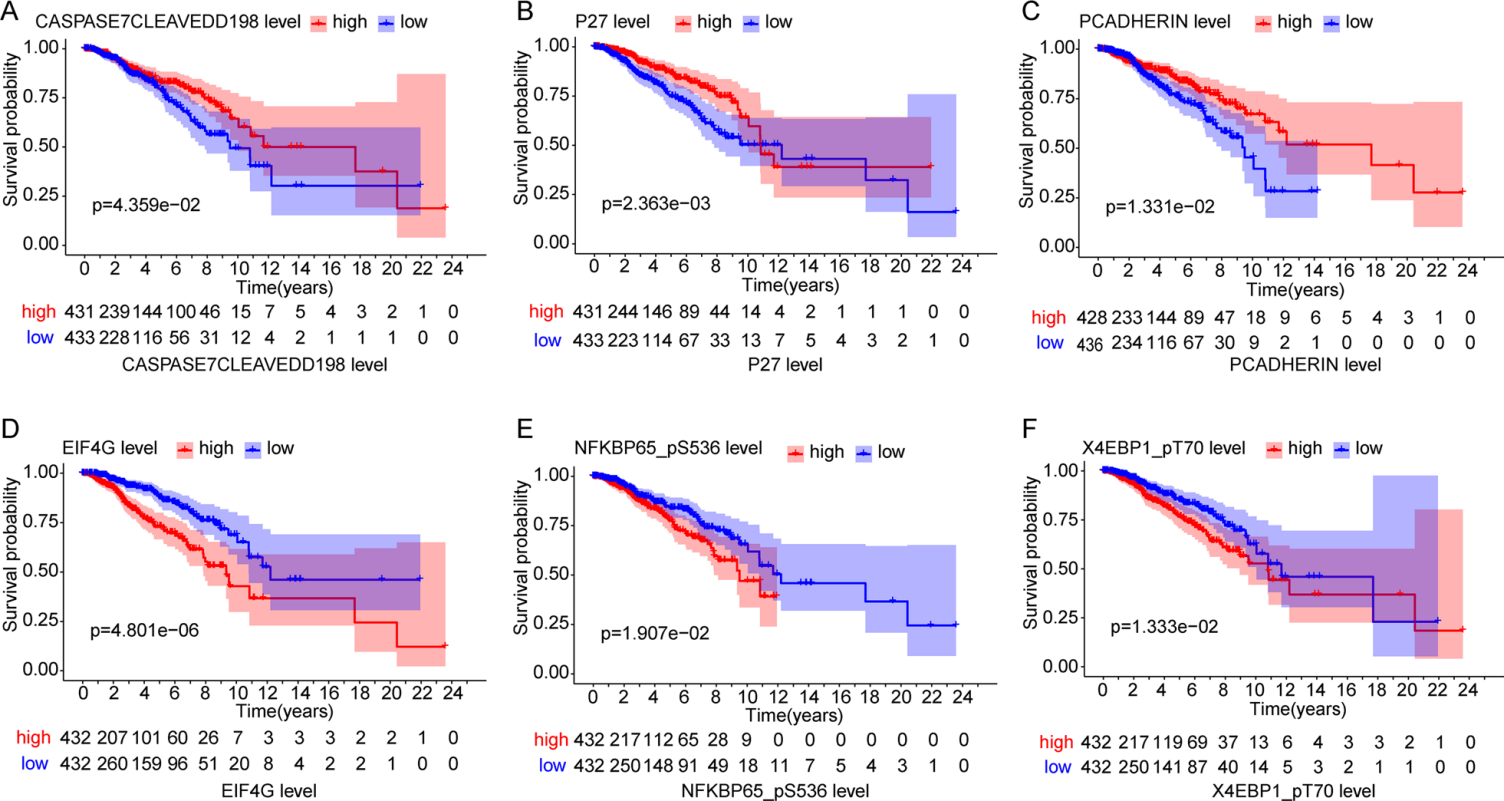
The relationship between the expression of 6 proteins and survival analysis of BRCA patients. The high expressive of CASPASE7CLEAVEDD198 (**A**), P27 (**B**), PCADHERIN (**C**) were better overall survival. The high expressive of EIF4G (**D**), NFKBP65_pS536 (**E**), X4EBP1_pT70 (**F**) were worse overall survival

To investigate the relationship between the expression of the 6 proteins and overall survival, we used the KM method to carry out survival analysis. According to the median value of protein expression, we divided all samples into high-expression and low-expression groups. The higher the protein expression levels were for CASPASE7CLEAVEDD198, P27, and PCADHERIN, the better the overall survival (Fig. 6A–C). The higher the protein expression levels were for EIF4G, NFKBP65-pS536, and X4EBP1-pT70, the poorer the overall survival (Fig. 6D–F).

### Differential expression analysis of the 6 proteins and their encoding genes

**Fig. 7:**
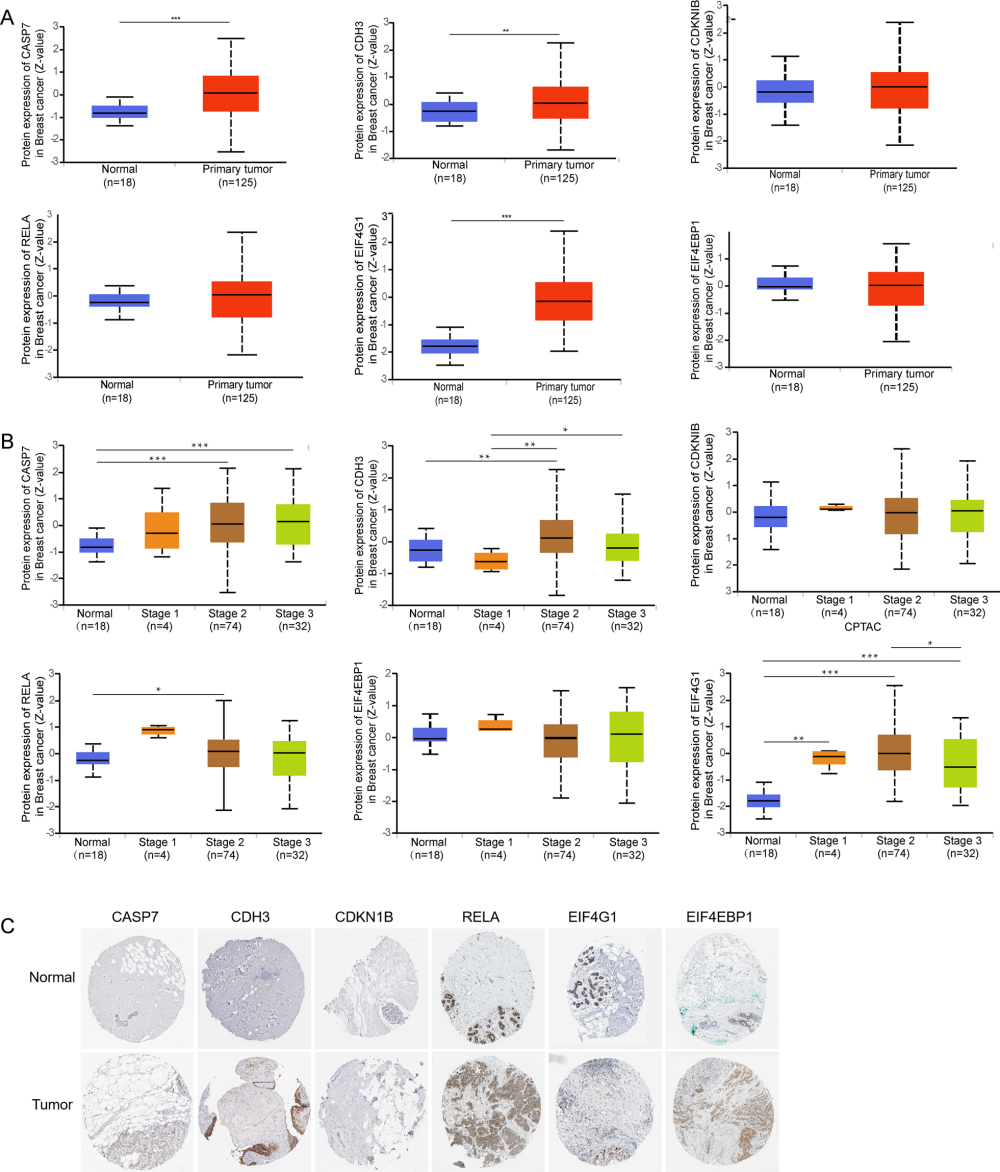
The expression of 6 proteins in BRCA. **A** The expression of 6 proteins in normal samples and tumor samples of BRCA. **B** The expression of 6 proteins in different stage of BRCA. **C** 6 proteins expressions in the Human Protein Atlas database

**Fig. 8:**
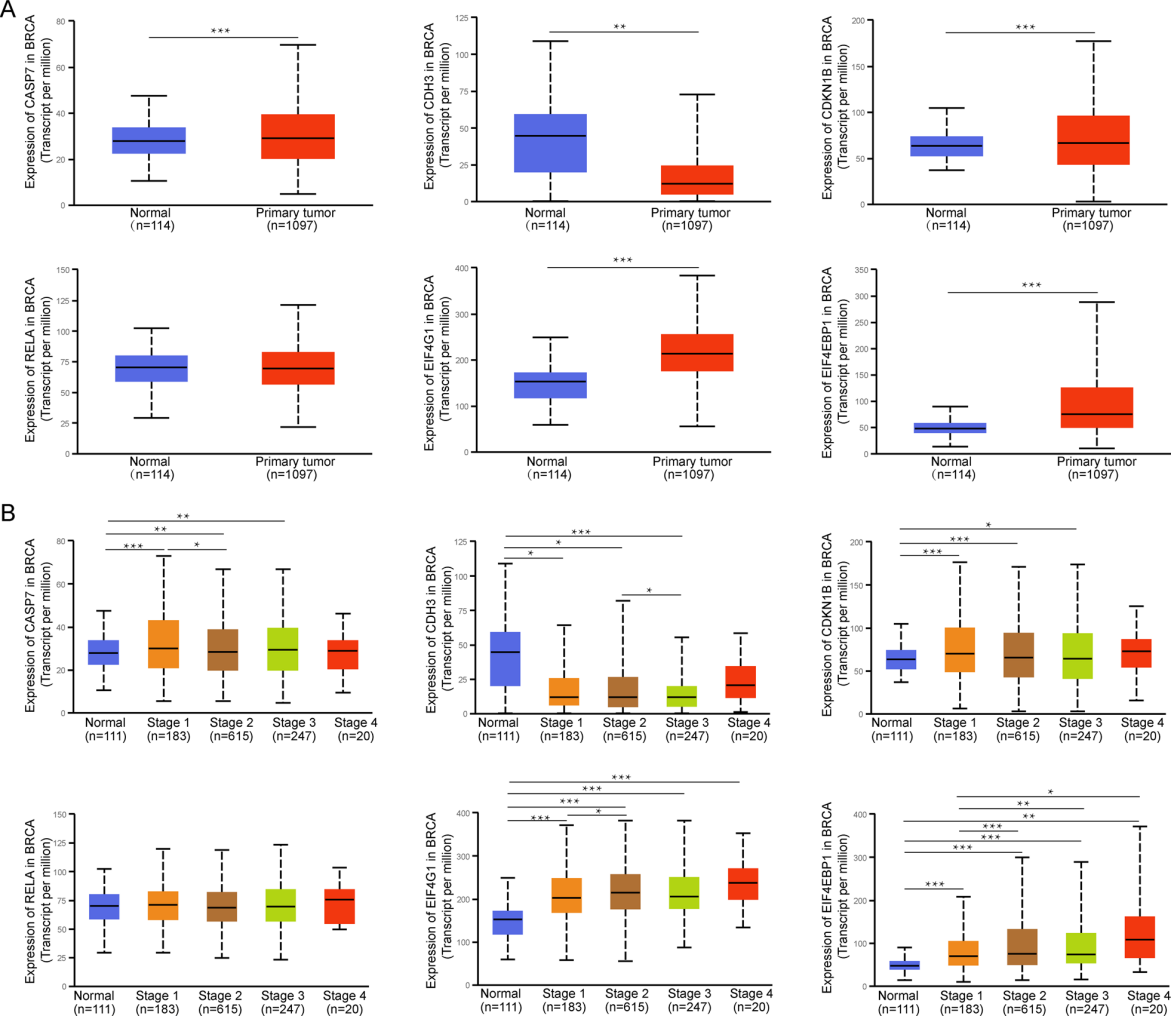
The expression of 6 encoding genes in BRCA. **A** The expression of 6 encoding genes in normal samples and tumor samples of BRCA. **B** The expression of 6 encoding genes in different stage of BRCA

To better understand the 6 prognosis-related proteins, we explored the expression of these proteins using the CPTAC dataset. The CASPASE7CLVEADD198 (CASP7), PCADHERIN (CDH3), and EIF4G (EIF4G1) were expressed at significantly higher levels in tumor tissue than in normal tissue, while P27 (CDKN1B), NFKBP65-pS536 (RELA), and X4EBP1-pT70 (EIF4EBP1) showed no difference (Fig. 7A). Except for CDKN1B and RELA, the expression levels of CASP7, CDH3, EIF4G1, and EIF4EBP1 were increased significantly in most individual cancer stages compared with normal samples (Fig. 7B). Moreover, we evaluated immunohistochemical images of these proteins from the Human Protein Atlas database (Fig. 7C) and the information of patients were shown in Additional file 1: Table S4. In addition, at the mRNA expression levels, there were differences in the expression of these encoding genes except RELA. Compared with normal samples, the expression level of CASP7, CDKN1B, EIG4G1, and EIF4EBP1 were significantly increased in the primary tumor samples, while that of CDH3 was significantly decreased (Fig. 8A). Similarly, except for RELA, the other encoding genes were expressed differently in the most individual cancer stages (Fig. 8B).

**Fig. 9:**
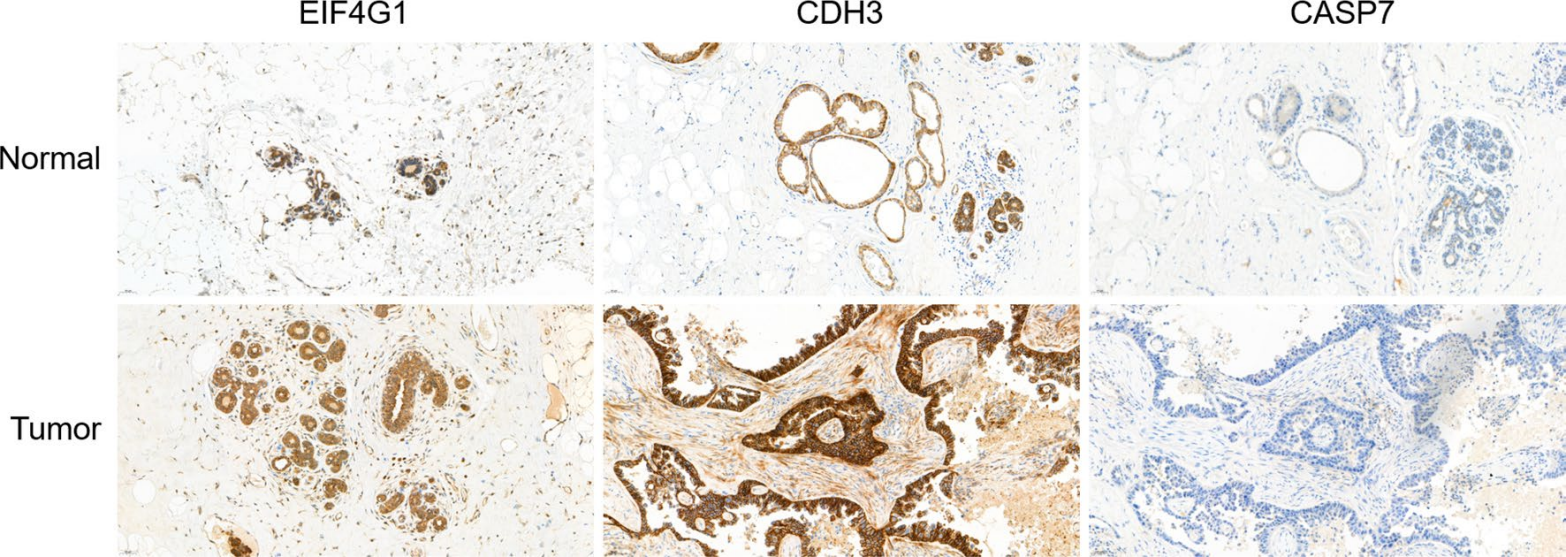
The expression of 3 differential proteins was analyzed by immunohistochemistry assay

To further confirm our analytical results, we used breast cancer tissues and paracancerous tissues to verify the expression of prognosis-related proteins with immunohistochemistry experiments. Pathology sections information was shown in Additional file 1: Tables S5 and S6. CDH3 and EIF4G1 were expressed at significantly higher levels in breast cancer tissues than in paracancerous tissues, but CASP7 was not (Fig. 9).

### Functional enrichment analysis

**Fig. 10:**
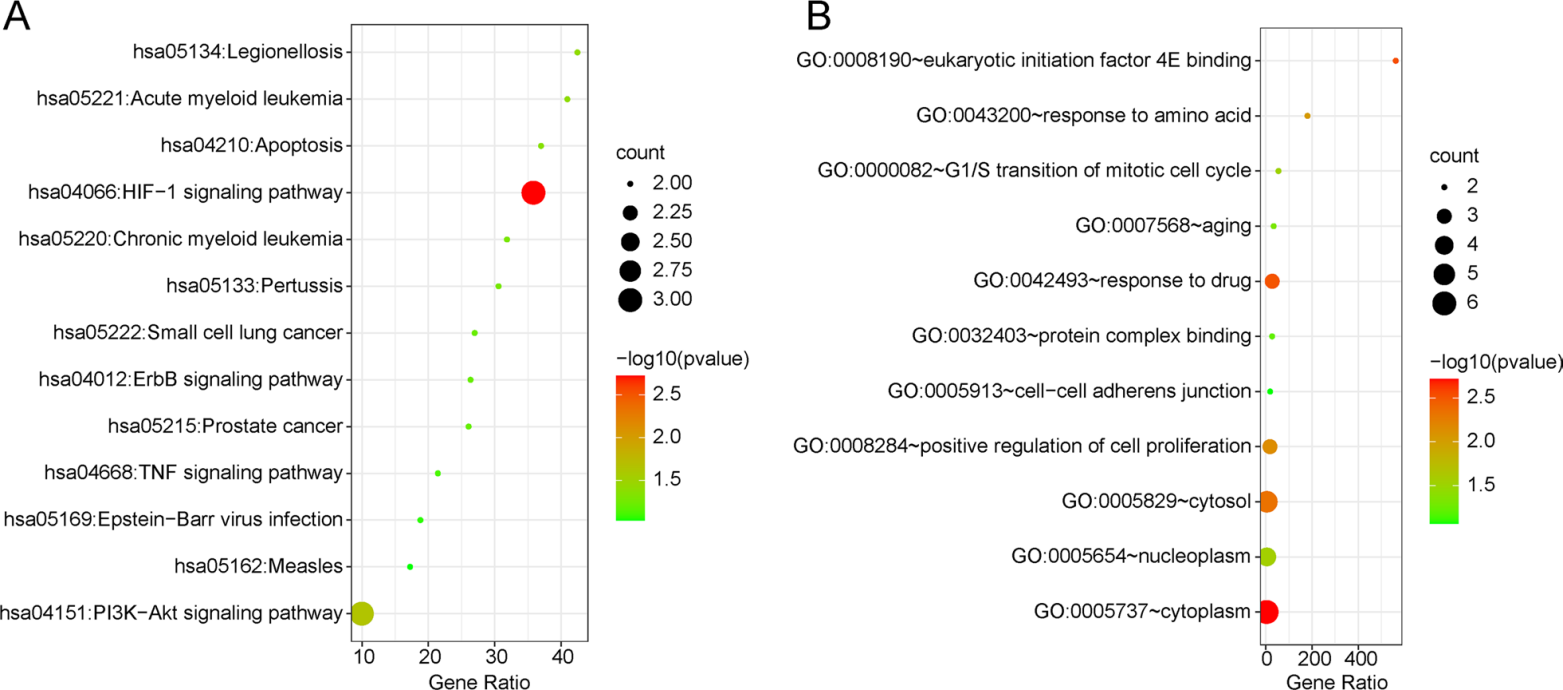
Functional enrichment analysis were performed by DAVID. **A** KEGG pathway analysis of 6 encoding genes. **B** GO analysis of 6 encoding genes

To determine the functions of these encoding genes, we performed the functional enrichment analysis by DAVID. KEGG pathway analysis indicated that these genes were mainly related to the HIF-1 and PI3K-AKT signaling pathways (Fig. 10A). GO analysis suggested that these genes were mainly located in the cytoplasm, cytosol, and nucleoplasm, and participated in certain biological processes, such as response to drug and positive regulation of cell proliferation (Fig. 10B).

## Discussion

Through combined analysis of the TCGA and TPCA datasets, cancer proteomics was used to study the pathogenesis and prognosis of tumors at the protein level and to explore candidate proteins that can be used as tumor biomarkers. This approach can provide new guidance for prognosis and personalized precision medicine of tumors. In this study, we obtained BRCA samples from the TCPA and TCGA datasets with clinical information and protein expression data. We screened six prognosis-related proteins using Kaplan–Meier and Cox analysis methods and further constructed a prognostic risk model using multivariate Cox analysis. These six prognosis-related proteins were PCADHERIN, CASPASE7CLVEADD198, P27, EIF4G, NFKBP65-pS536, and X4EBP1-pT70, which were remarkably correlated with the overall survival of patients with BRCA. We assessed the effectiveness of the prognostic risk model in the GSE88770 dataset by survival analysis and ROC curve analyses. This study indicated that the prognostic risk model could serve as a sensitive independent prognostic factor and effectively predict the prognosis of BRCA patients.

Studies have shown that some of the prognosis-related proteins are involved in the prognosis of malignancy. Caspase-7 is the main executioner of mitochondrial apoptosis. Apoptosis maintains the homeostasis balance between cell proliferation and cell death. The dysregulation of apoptosis can lead to cancer [35]. As an indicator of apoptosis impairment, high cleaved caspase-7 levels indicate a decreased risk in BRCA and may be an alternative prognostic biomarker of clinical outcome [36]. In colorectal cancer (CRC) patients, the upregulation of caspase-7 caused by downregulation of sterol regulatory element binding proteins (SREBP1) may improve chemosensitivity to gemcitabine in CRC cells, which may serve as a novel prognostic biomarker for CRC [37]. This is basically consistent with the results of our studies. High expression of caspase-7 is associated with a good prognosis. Placental-cadherin (CDH3) is a cell adhesion molecule that has different roles in different tumors. CDH3 is highly expressed and promotes tumorigenesis in pancreatic cancer, gastric cancer, and breast cancer, while it is expressed at low levels and suppresses tumorigenesis in non-small-cell lung cancer, hepatocellular carcinoma and thyroid cancer [38–41]. P27 is an atypical tumor suppressor that regulates the cell cycle, cell migration and development and plays both tumor-suppressive and oncogenic roles [42, 43]. Deletion or mutational inactivation in the p27 gene is rare in human cancers. However, excessive proteolysis of p27 results in loss of growth restraint function in human cancers [44]. In PI3K/AKT activated cancers, C-terminally phosphorylated p27 is overexpressed in the cytoplasm and nucleus, and binds proteins to drive tumor progression, which shifts p27 from a cyclin-dependent kinase inhibitor to an oncogene [45]. In PI3K/AKT-activated human breast cancers, highly stable p27 accumulates in the cytoplasm and increases tumor metastasis, which is associated with poor patient outcome [46]. p27 is a key target of miR-221/222 in triple-negative breast cancer [47]. In our study, the expression of p27 was positively correlated with prognosis. NF-κB is a family of ubiquitous transcription factors that regulate DNA transcription, cytokine production, and cell survival [48]. The NF-κB signaling pathway plays an important role in inflammation and the immune response. Inflammatory cytokines can drive NF-κB activation within the tumor microenvironment. In solid tumors, NF-κB is activated and promotes cancer cell growth and metastasis in breast, pancreatic, and colorectal cancers [49–51]. RELA/p65 is an important subunit of the NF-κB family, and Ser536 is an important phosphorylation site in RELA/p65. The phosphorylation of p65 at Ser536 was upregulated with the maturation and apoptotic shedding of epithelial cells in normal colon mucosa but was downregulated in colon cancer. In colon, breast, and prostate cancer cells, the RELA/p65 phosphomimetic mutation at Ser536 triggered dramatic apoptosis and suppressed tumor growth by affecting the expression of genes related to cell death or survival in nude mice [52]. In our study, the expression of RELA/p65 showed no significant difference between normal breast tissue and tumor breast tissue. The phosphorylation of p65 at Ser536 is a high-risk protein in BRCA. EIF4G1 is the major isoform of the EIF4G family and is a critical component of the eukaryotic initiation factor (EIF)4F complex. EIF4G1 is required for cap-dependent mRNA translation which is a necessary process for tumor growth and survival. Studies suggest that EIF4G1 is overexpressed in several solid tumors and plays an important role in the tumorigenesis. High expression of EIF4G1 has been found in various tumors and is associated with poor prognosis, including breast, lung, hypopharyngeal, and nasopharyngeal cancers [53–55], which is consistent with our findings. Recent studies suggest that high expression of EIF4G1 is associated with poor prognosis of pancreatic ductal adenocarcinoma and prostate cancer and may serve as a novel prognostic biomarker [56, 57]. Based on previous studies, these prognosis**-**related proteins play an important role in various tumors. Our study further reveals the prognostic roles of these proteins in BRCA.

## Conclusions

In summary, we constructed and assessed a prognostic risk model based on these proteins for BRCA patients. There are few protein databases that can be used publicly and contain relevant clinical information at present. Therefore, we chose GSE database to verify the prognostic model at the mRNA levels. The validate results showed that this prognostic model had good predictive performance and could be used to predict the risk of BRCA patients, which could explain the role of the protein prognosis model to a certain extent. The prognostic risk model will provide new insight into the diagnosis and prognosis of BRCA. In the future, we will continue to collect clinical samples and explore new databases for a more comprehensive validation.

## Supplementary Information

**Additional file 1: Supplementary Table 1.** Low-risk and high-risk proteins associated with the survival of BRCA patients. **Supplementary Table 2.** Clinical characteristics of BRCA patients included in this study. **Supplementary Table 3.** Clinical characteristics of BRCA patients in the GSE88770 dataset. **Supplementary Table 4.** The immunohistochemical images information of these proteins in the Human Protein Atlas database. **Supplementary Table 5.** Clinicopathological features of 20 BRCA patients. **Supplementary Table 6.** The EIF4G1, CDH3, CASP7 expression level between tumor and para-tumor tissues.

## Data Availability

Publicly available datasets were analyzed in this article. These data can be found here: The TCPA database (http://www.tcpaportal.org/tcpa/). The TCGA database (http://portal.gdc.cancer.gov/). The Gene Expression Omnibus database (GEO, https://www.ncbi.nlm.nih.gov/geo/).
