# Supplementary material for: Construction and validation of a prognostic risk model for breast cancer based on protein expression
Source: BMC Med Genomics. 2022 Jul 4;15:148. doi: 10.1186/s12920-022-01299-5 (PMC9252042; doi:10.1186/s12920-022-01299-5)
Supplement: Supplementary file 1 — Additional file 1: Supplementary Table 1. Low-risk and high-risk proteins associated with the survival of BRCA patients. Supplementary Table 2. Clinical characteristics of BRCA patients included in this study. Supplementary Table 3. Clinical characteristics of BRCA patients in the GSE88770 dataset. Supplementary Table 4. The immunohistochemical images information of these proteins in the Human Protein Atlas database. Supplementary Table 5. Clinicopathological features of 20 BRCA patients. Supplementary Table 6. The EIF4G1, CDH3, CASP7 expression level between tumor and para-tumor tissues. [file 12920_2022_1299_MOESM1_ESM.docx]

Supplementary Material

**Construction and validation of a** **prognostic risk model for breast cancer based on protein expression**

Bo Huang ^1†^, Xujun Zhang^2†^, Qingyi Cao^2^, Jianing Chen^2^, Chenhong Lin^3^, Tianxin Xiang^4^, Ping Zeng^4*^

^1^ Department of Gynecology and Obstetrics, The First Affiliated Hospital, Zhejiang University School of Medicine, Hangzhou, China.

^2^ State Key Laboratory for Diagnosis and Treatment of Infectious Diseases National Clinical Research Center for Infectious Diseases, National Medical Center for Infectious Diseases, Collaborative Innovation Center for Diagnosis and Treatment of Infectious Diseases, The First Affiliated Hospital, Zhejiang University School of Medicine, Hangzhou, China.

^3^ Department of Gastroenterology, Sir Run Run Shaw Hospital, Zhejiang University School of Medicine, Hangzhou, China

^4^ Department of Hospital Infection Control, The First Affiliated Hospital of Nanchang University, Nanchang, China.

***Correspondence:**

Ping Zeng

**Address:** Department of Hospital Infection Control, The First Affiliated Hospital of Nanchang University. 17 Yongwai Road, Donghu District. Nanchang, China.

**Tel:** +86 15879193240

**Email:** ndyfy08004@ncu.edu.cn

† These authors have contributed equally to this work and share authorship.

| **Additional file 1: Supplementary Table 1** Low-risk and high-risk proteins associated with the survival of BRCA patients. | | | | | |
| --- | --- | --- | --- | --- | --- |
| Low-risk Proteins | | | | | |
| Gene | KM | HR | HR.95L | HR.95H | P value |
| DVL3 | 0.0011453 | 3.2064409 | 1.8029946 | 5.702326 | 7.29E-05 |
| EIF4G | 4.80E-06 | 2.2800379 | 1.453756 | 3.5759597 | 0.0003314 |
| X4EBP1 | 0.1204944 | 1.5563041 | 1.1937981 | 2.0288879 | 0.0010783 |
| RB_pS807S811 | 0.011313 | 1.6834261 | 1.2225616 | 2.3180209 | 0.0014168 |
| NFKBP65_pS536 | 0.0190715 | 1.4984838 | 1.1666967 | 1.924625 | 0.0015384 |
| PAXILLIN | 0.017882 | 2.1661234 | 1.3065715 | 3.5911473 | 0.0027291 |
| TUBERIN | 0.0758338 | 2.1382475 | 1.2790282 | 3.574669 | 0.0037484 |
| X4EBP1_pT70 | 0.0133324 | 2.6091785 | 1.3259579 | 5.1342598 | 0.0054882 |
| GSK3_pS9 | 0.0540011 | 1.5357083 | 1.1216846 | 2.1025519 | 0.0074425 |
| CIAP | 0.3201839 | 3.0923045 | 1.3468504 | 7.0997844 | 0.0077644 |
| GSK3ALPHABETA_pS21S9 | 0.0344991 | 1.4842585 | 1.0944205 | 2.0129589 | 0.0110742 |
| X4EBP1_pS65 | 0.0051129 | 1.8786248 | 1.1490661 | 3.071391 | 0.0119386 |
| TUBERIN_pT1462 | 0.0463456 | 1.9064795 | 1.0811252 | 3.3619271 | 0.0257823 |
| IRS1 | 0.2301233 | 1.7042251 | 1.0647779 | 2.7276892 | 0.026316 |
| SLC1A5 | 0.0796804 | 1.3708115 | 1.0199419 | 1.8423836 | 0.0365405 |
| GAB2 | 0.0368029 | 1.353213 | 1.0115049 | 1.8103576 | 0.0416502 |
| CLAUDIN7 | 0.1847533 | 1.2699447 | 1.0028981 | 1.6080992 | 0.047257 |
| PRAS40_pT246 | 0.1108034 | 2.2718103 | 1.0057653 | 5.1315369 | 0.0484054 |
| High-risk Proteins | | | | | |
| Gene | KM | HR | HR.95L | HR.95H | P value |
| LCK | 0.0017632 | 0.4956542 | 0.3337127 | 0.7361813 | 0.0005063 |
| PCADHERIN | 0.0133059 | 0.2199207 | 0.0908203 | 0.5325363 | 0.0007897 |
| P27 | 0.0023633 | 0.4235691 | 0.2449278 | 0.7325045 | 0.0021135 |
| COLLAGENVI | 0.0120077 | 0.6941578 | 0.5477917 | 0.8796319 | 0.0025155 |
| HEREGULIN | 0.5812761 | 0.2361306 | 0.0852639 | 0.6539427 | 0.0054829 |
| STAT3_pY705 | 0.0069015 | 0.5234557 | 0.315307 | 0.869013 | 0.012321 |
| CASPASE8 | 0.0110439 | 0.3021686 | 0.1168387 | 0.7814692 | 0.0135648 |
| CASPASE7CLEAVEDD198 | 0.0435893 | 0.695975 | 0.5171852 | 0.936572 | 0.0167327 |
| X1433EPSILON | 0.0312498 | 0.2560763 | 0.0815383 | 0.8042245 | 0.0196423 |
| MYH11 | 0.1083758 | 0.8536696 | 0.7428375 | 0.9810379 | 0.0257624 |
| SETD2 | 0.0680439 | 0.3788174 | 0.1567274 | 0.915619 | 0.0311041 |
| IGF1R_pY1135Y1136 | 0.1095361 | 0.3824317 | 0.1550588 | 0.9432163 | 0.0368984 |
| ACVRL1 | 0.179912 | 0.392119 | 0.1592121 | 0.9657393 | 0.0417734 |
| PEA15 | 0.1665716 | 0.5428714 | 0.2978543 | 0.9894414 | 0.046084 |
| HSP70 | 0.1605874 | 0.8073819 | 0.6537029 | 0.9971892 | 0.0470231 |
| NRAS | 0.4656432 | 0.4447041 | 0.1988775 | 0.9943895 | 0.0484196 |

**Additional file 2: Supplementary Table 2** Clinical characteristics of BRCA patients included in this study

| Clinical characteristics | No. of patients (%) |
| --- | --- |
| Age |  |
| ≤58 | 472 (52.27%) |
| >58 | 431 (47.73%) |
| Stage |  |
| Ⅰ | 160 (17.72%) |
| Ⅱ | 527 (58.36%) |
| Ⅲ | 200 (22.15%) |
| Ⅳ | 16 (1.77%) |
| T |  |
| 1 | 235 (26.02%) |
| 2 | 533 (59.03%) |
| 3 | 103 (11.41%) |
| 4 | 32 (3.54%) |
| M |  |
| 0 | 887 (98.23%) |
| 1 | 16 (1.77%) |
| N |  |
| 0 | 448 (49.61%) |
| 1 | 298 (33%) |
| 2 | 102 (11.30%) |
| 3 | 55 (6.09%) |

**Additional file 3: Supplementary Table 3** Clinical characteristics of BRCA patients in the GSE88770 dataset.

| Clinical characteristics | No. of patients (%) |
| --- | --- |
| Status |  |
| Alive | 89 (76.07) |
| Death | 28 (23.93) |
| Subtype |  |
| Alveolar | 3 (2.54) |
| Classic | 86 (72.88) |
| Mixed | 3 (2.54) |
| Solid | 11 (9.32) |
| Trabecular | 15 (12.71) |
| ER |  |
| Positive | 106 (90.60) |
| Negative | 11 (9.40) |
| PR |  |
| Positive | 79 (68.10) |
| Negative | 37 (31.90) |
| HER2 |  |
| Positive | 7 (6.09) |
| Negative | 108 (93.91) |
| Grade |  |
| Grade 1 | 13 (11.21) |
| Grade 2 | 96 (82.76) |
| Grade 3 | 7 (6.03) |

**Additional file 4: Supplementary Table 4** The immunohistochemical images information of these proteins in the Human Protein Atlas database

|  | CASP7  (CAB025563) ^a^ | |  | CDH3  (CAB025563) ^a^ | |  | CDKNIB  (CAB025563) ^a^ | |  | RELA  (CAB025563) ^a^ | |  | EIF4G1  (CAB025563) ^a^ | |  | EIF4EBP1  (CAB025563) ^a^ | |
| --- | --- | --- | --- | --- | --- | --- | --- | --- | --- | --- | --- | --- | --- | --- | --- | --- | --- |
|  |  |  |  |  |  |  |  |  |  |  |  |  |  |  |  |  |  |
|  | N | T |  | N | T |  | N | T |  | N | T |  | N | T |  | N | T |
| Patient id  (FEMALE) | 2259 | 1785 |  | 2042 | 315 |  | 1801 | 2018 |  | 2773 | 2073 |  | 3286 | 2392 |  | 3158 | 2091 |
| Age | 23y | 93y |  | 75y | 50y |  | 39y | 51y |  | 23y | 51y |  | 27y | 27y |  | 52y | 40y |
| **a**: Antibody Catalog # | | | | | | | | | | | | | | | | | |

**Additional file 5: Supplementary Table 5** Clinicopathological features of 20 BRCA patients

| Pathological parameters | No. of patients (%)  n=20 |
| --- | --- |
|  |  |
|  |  |
| Age |  |
| ＞40 | 9 (45) |
| ≤40 | 11 (55) |
| Tumour type |  |
| Ductal | 14 (70) |
| Lobular | 4 (20) |
| Other | 2 (10) |
| Tumour grade |  |
| 1 | 10 (50) |
| 2 | 6 (30) |
| 3 | 4 (20) |
| TNM stage |  |
| T1 | 8 (40) |
| T2 | 7 (35) |
| T3 | 3 (15) |
| T4 | 2 (10) |
| Clinical staging |  |
| Ⅰ | 9 (45) |
| Ⅱ | 6 (30) |
| Ⅲ | 3 (15) |
| Ⅳ | 2 (10) |

**Additional file 6: Supplementary Table 6** The EIF4G1, CDH3, CASP7 expression level between tumor and para-tumor tissues

| Samples | n=20 | EIF4G1 | | p-value | CDH3 | | p-value | CASP7 | | p-value |
| --- | --- | --- | --- | --- | --- | --- | --- | --- | --- | --- |
|  |  | Low | High |  | Low | High |  | Low | High |  |
| Tumor | 20 | 4 | 16 | 0.011 | 3 | 17 | 0.001 | 19 | 1 | 0.598 |
| Para-tumor | 20 | 13 | 7 |  | 14 | 6 |  | 17 | 3 |  |
| p-value: χ2 test. | |  |  |  |  |  |  |  |  |  |
